# Adverse drug reactions and events in an Ageing PopulaTion risk Prediction (ADAPTiP) tool: the development and validation of a model for predicting adverse drug reactions and events in older patients

**DOI:** 10.1007/s41999-024-01152-1

**Published:** 2025-01-17

**Authors:** Juliane Frydenlund, Nicole Cosgrave, Frank Moriarty, Emma Wallace, Ciara Kirke, David J. Williams, Kathleen Bennett, Caitriona Cahir

**Affiliations:** 1 Data Science Centre, School of Population Health, RCSI University of Medicine and Health Science, Lower Mercer Street, Dublin 2, Ireland; 2 Department of Geriatric and Stroke Medicine, RCSI University of Medicine and Health Sciences, Dublin, Ireland; 3 School of Pharmacy and Biomolecular Sciences, RCSI University of Medicine and Health Sciences, Dublin, Ireland; 4 Department of General Practice, School of Medicine, University College Cork, Cork, Ireland; 5 National Quality and Patient Safety Directorate at Health Service Executive, Dublin, Ireland

**Keywords:** Older populations, Adverse drug reaction (ADR), Adverse drug event (ADE), Risk prediction, High-risk prescribing

## Abstract

**Purpose:**

Older people are at an increased risk of developing adverse drug reactions (ADR) and adverse drug events (ADE). This study aimed to develop and validate a risk prediction model (ADAPTiP) for ADR/ADE in older populations.

**Methods:**

We used the adverse drug reactions in an Ageing PopulaTion (ADAPT) cohort (*N* = 798; 361 ADR-related admissions; 437 non-ADR-related admissions), a cross-sectional study designed to examine the prevalence and risk factors for ADR-related hospital admissions in patients aged ≥ 65 years. Twenty predictors (categorised as sociodemographic-related, functional ability-related, disease-related, and medication-related) were considered in the development of the model. The model was developed using multivariable logistic regression and was internally validated by fivefold cross-validation. The model was externally validated in a separate prospective cohort from the Centre for Primary Care Research (CPCR) study of ADES. The cross-validated and externally validated model performance was evaluated by discrimination and calibration.

**Results:**

The final prediction model, ADAPTiP, included nine predictors: age, chronic lung disease, the primary presenting complaints of respiratory, bleeding and gastrointestinal disorders and syncope on hospital admission and antithrombotics, diuretics, and renin–angiotensin–aldosterone system drug classes. ADAPTiP demonstrated good performance with cross-validated area under the curve of 0.75 [95% CI 0.72;79] and 0.83 [95% CI 0.80;0.87] in the external validation.

**Conclusion:**

Using accessible information from medical records, ADAPTiP can help clinicians to identify those older people at risk of an ADR/ADE who should be monitored and/or have their medications reviewed to avoid potentially harmful prescribing.

**Supplementary Information:**

The online version contains supplementary material available at 10.1007/s41999-024-01152-1.

## Key summary points

**Aim:**

To develop and validate a risk prediction model for ADR/ADE in older populations based on easily accessible data for clinicians.

**Findings:**

Based on accessible information for clinicians this model demonstrated good performance with cross-validated area under the curve of 0.75 [95% CI 0.72;79] and 0.83 [95% CI 0.80;0.87] in the external validation.

**Message:**

This prediction model, ADAPTiP, can help clinicians identify older people at risk of an ADR/ADE who should have their medications reviewed to avoid potentially harmful prescribing.

**Supplementary Information:**

The online version contains supplementary material available at 10.1007/s41999-024-01152-1.

## Introduction

The complexities of ageing and its innate changes in pharmacokinetics and pharmacodynamics in addition to increased frailty, decompensation, co-morbidities, and medication utilisation can increase the risk of medication-related harm, including adverse drug reactions (ADRs) and adverse drug events (ADE) in older populations [1, 2]. Medication-related harm has been identified internationally as a key area for improvement and the World Health Organisation (WHO) third global patient safety challenge aims to promote and implement actions for reducing the number of preventable ADRs and ADEs [3]. Accurately predicting ADRs and ADEs in older populations is challenging given the numerous risk factors contributing to ADR and ADE occurrence and the heterogeneity of older populations [4]. However, efforts need to continue to develop practical and efficient tools to identify older patients at increased risk of ADRs and ADEs and to reduce medication-related harm morbidity and costs in our growing ageing population.

ADRs are described as ‘an appreciably harmful or unpleasant reaction resulting from an intervention relating to the use of a medicinal product’, while ADEs are a broader term for injuries related to medicine use [4, 5]. Studies have reported that approximately 10% of hospital admissions are ADR-related older populations and approximately 70% of these admissions are potentially avoidable, with the average cost estimated as €9538 (SD €10,442) per ADR-related hospital admission [6–8]. Similarly, 25–50% of ADEs have been shown to be potentially detectable and mitigatable at an early stage in outpatient settings [9].

Risk prediction tools would enable clinicians to identify those patients who may require monitoring and review of their medications to avoid potentially harmful prescribing cascades (e.g., a new medication being started to manage a potentially unrecognised ADR/ADE) [10]. However, to date, the detection and prediction of ADRs/ADEs remain somewhat elusive. There is a lack of robust risk prediction models which are externally validated and demonstrate good performance [area under the receiver-operator curve (ROC) ranging from 0.62 to 0.73 in previous models] and which are feasible for routine use in clinical practice [11]. There is some evidence that increasing age, polypharmacy, multimorbidity, prior ADR/ADE, and dementia in the acute setting are associated with ADRs/ADEs, but the predictive factors are still poorly understood, particularly in the community setting [12].

There is also evidence that current approaches/methods for predicting and detecting ADRs and ADEs in older patients are insufficient and newer approaches should be adopted that focus on the ADR/ADE risk associated with particular drugs, drug classes or clinical syndromes [13]. ADR/ADE risk prediction tools need to be practical, efficient, and easy to apply to be of use in clinical practice. They also need to be focussed on specific risk factors which are easily identifiable for healthcare practitioners. The aim of this study was to develop and externally validate a risk prediction model with the title Adverse Drug reactions and events in an Ageing PopulaTion risk Prediction (ADAPTiP) tool, for prediction of ADR\ADEs in older community-based populations.

## Methods

The Transparent Reporting of a multivariable prediction models for Individual Prognosis Or Diagnosis (TRIPOD) guidelines were used to develop the risk prediction tool of this study [14] (Supplementary Table S1). Geographical validation was used to externally validate the risk prediction model and in particular domain or setting validation. Geographical validations studies are a type of external validation where different in/exclusion criteria, and predictor and outcome definitions and measurements, are applied and compared to the development study. Domain or setting validation is a particular rigid form of geographical validation where the developed model is validated in very different individuals, e.g., validating a prediction model developed in a cohort of older community-based people being admitted acutely to hospital with a potential ADR (or not) in a cohort of older community-based patients experiencing a potential ADE (or not) in primary care [15].

### Sources of data and participants

In this study, two sources of data were used: one dataset for the development and internal validation of the prediction model, and another dataset to externally validate the prediction model in a different setting [16]. For the development of the prediction model, we used the Adverse Drug reactions in an Ageing PopulaTion (ADAPT) cohort (*N* = 798). ADAPT is a cross-sectional study designed to examine the prevalence and risk factors for ADR-related hospital admissions, in all community-based patients aged ≥ 65 years admitted acutely to a large tertiary referral hospital in Ireland over an 8-month period (November 2016–June 2017) [6, 17]. The ADAPT database contains details of the patients’ prescriptions and their medical record and a range of potential predictors for ADRs, based on previous systematic reviews. A protocol and detailed description of the ADAPT cohort has been previously published [6, 17].

For external validation of the prediction model, we used the Centre for Primary Care Research (CPCR) prospective cohort. The CPCR cohort is a prospective cohort study of general practice (GP) patients aged ≥ 70 years recruited from 15 practices in Ireland [Wave 1 (2010–2011)–Wave 2 (2012–2013)]. The CPCR database contains data from patients' medical records, ADEs, a self-reported patient questionnaire measuring potential predictors for ADEs, linked to prescription dispensing information from the national Health Service Executive-Primary Care Reimbursement Service (HSE-PCRS) General Medical Services (GMS) scheme. A detailed description of the CPCR cohort has been previously published [18, 19].

### Outcome: ADR and ADE

Within the ADAPT cohort used for the development of the model (*N* = 798), 361 patients were determined to have an ADR-related admission (14). ADR-related admissions were determined using a multifaceted review of each hospital admission to assess the likelihood of the ADR being a reason for admission (cause of admission or contributing to admission) in the context of the patient’s medication, clinical conditions, medical history, co-morbidities, and investigations, and using validated algorithms and decision aids [20–22]. Within the same cohort, 437 patients were determined not to have a suspected ADR at hospital admission (control group for comparative purposes). A detailed analysis of the prevalence and nature of the ADR-related hospital admissions (causality, severity, and preventability) within the ADAPT cohort has been published previously [6, 17].

In the CPCR dataset used for external validation of the prediction model (*N* = 605), 445 patients were determined to have an ADE (*N* = 160 non-ADE group). ADEs detected in the CPCR cohort were primarily mild and few resulted in hospital admission. An ADE was determined through patient interviews and review of GP medical records in the previous 6 months. All patient-reported ADEs were independently reviewed by two academic GPs, who rated the likelihood of each patient-reported ADE being a true ADE on a Likert 6-point scale (1 = no confidence to 6 = certain) [23]. Only ADEs where both reviewers rated the ADE as likely (≥ 50% likelihood; score ≥ 4) were included. A detailed analysis of the prevalence and nature of the ADEs within the CPCR cohort has been published previously [19].

### Potential predictors

A number of potential predictors of an ADR-related hospital admission were measured as part of the ADR screening process on hospital admission within the ADAPT cohort. The following 20 predictors were considered in the development of the risk prediction model and were categorised as: sociodemographic-related predictors (age, gender (female/male)); functional ability-related predictors/geriatric syndromes (immobility (Yes/No), self-reported history of falls (yes/no), frailty (Triage Risk Screening (TRS) tool (yes/no) [24] and delirium (4AT score)); disease-related predictors (chronic lung disease, primary presenting complaint at hospital admission (respiratory disorders, bleeding disorders, gastrointestinal disorders, syncope) and Charlson co-morbidity index (0 points, 1–2 points, and ≥ 3 points)); and medication-related predictors based on drug classes (antithrombotic agents (ATC: B01), diuretics (ATC: C03), agents acting on the renin–angiotensin–aldosterone system (RAAS) (ATC: C09), calcium channel blockers (ATC: C08), beta-blocking agents (ATC: C07), psychoanaleptics (ATC: N06), non-steroidal anti-inflammatory drugs (NSAID) (ATC: M01), and polypharmacy (0–4 (non-polypharmacy), 5–9 (polypharmacy), 10 + (significant polypharmacy)) [25]. These predictors were considered in the development of the model as they had been found to be either; (1) significantly associated with ADR-related hospital admissions in the ADAPT cohort per previous research [17]; or (2) there was strong evidence of their association with ADR-related hospital admissions as per previous systematic reviews of risk factors for ADRs/ADEs (gender, co-morbidity, polypharmacy, and NSAIDs) [7, 12] or existing literature regarding frailty [26, 27].

### Sample size

To compute the minimum sample size required for the development of a new multivariable prediction model, we used the criteria proposed by Riley et al. [28]. Overall, prevalence of ADRs was 45% of the study population. The number of candidate predictor parameters for potential inclusion in the new model was 10. The C-statistic was estimated at 0.7. This gave a minimum sample size of 701. This criterion is thereby fulfilled in this study.

### Data analysis

Univariate logistic regression was used to estimate unadjusted odds ratios (OR) and 95% confidence intervals (CI) for associations between each of the 20 potential predictors and ADR-related hospital admissions. Multivariable logistic regression with stepwise backward selection was performed [29, 30]. Adjusted odds ratios (AOR) and 95% CIs were estimated for all included predictor variables. To limit collinearity and ensure a parsimonious model, Spearman's correlations were calculated between predictors that were considered to be clinically related. The final model was identified based on the lowest value of the Bayesian Information Criterion (BIC).

Estimates of stepwise area under the receiver-operator curve (AUC) were calculated for the selected predictors in the final model. The stepwise AUCs were calculated by a hierarchical approach, where the predictors in the final model were added step by step, starting with the most significant predictor. Internal validation of the model was performed using fivefold cross-validation where the study cohort was randomly divided into five equally sized folds (subsamples). Then, each of the fivefold were in turn left out of the renewed model building and used as a validation cohort instead. The model was then externally validated in the CPCR cohort by identifying the same predictors and applying the weights calculated in the ADAPT development dataset.

The cross-validated and externally validated model performance was evaluated by discrimination and calibration. Discrimination between patients with and without an ADR (ADAPT dataset) and with and without an ADE (CPCR dataset) were calculated and displayed as a ROC and presented as AUC. AUC of less than 0.5 indicates poor discriminative ability, while a value of 1 represents perfect discriminative ability [31]. In general, AUC ≥ 0.75 are determined to be clinically useful [32]. Calibration, measuring the agreement between observed and predicted probabilities was assessed visually for both the cross-validated and externally validated model. Missing values (< 7%) were assumed missing at random. All statistical analysis was undertaken using Stata Version 18.0 (StataCorp, College Station, TX, United States). Significance was defined as a two-tailed *p* < 0.05.

## Results

### Characteristics of data sources and participants

The median age of the development dataset (ADAPT *N* = 798) was 81 years [IQR: 75–86], with 256 (32%) patients aged over 85 years. On average, patients were prescribed 11 [IQR: 7–14] different drug classes, and 324 (41%) patients had a Charlson Comorbidity Index score ≥ 3. In the external validation dataset (CPCR *N* = 605), the median age was 79 [IQR: 75;83]. On average patients were prescribed 6 (IQR 4,8) different drug classes and 91 (15%) patients had a Charlson Comorbidity Index score ≥ 3.

### Potential predictors

Table 1 presents the univariate associations for the 20 potential predictors with ADR-related hospital admissions and non-ADR-related hospital admissions in the development dataset (ADAPT). Presenting with complaints of, bleeding disorders, gastrointestinal disorders, and syncope and being prescribed antithrombotic agents, diuretics and renin–angiotensin–aldosterone system (RAAS) drug classes were significantly associated with ADR-related hospital admissions. Increasing age, presenting with a respiratory complaint, and having chronic lung disease were significantly associated with not having an ADR-related hospital admission. These predictors were all considered in the multivariable logistic regression models.

**Table 1:** Unadjusted odds ratios (OR) and 95% CI for potential risk predictor associations with ADR-related hospital admissions and non-ADR-related hospital admissions (*N* = 798)

Potential risk predictors	ADR-related hospital admissions * N* = 361	Non ADR-related hospital admissions * N* = 437	Unadjusted OR [95% CI]
Sociodemographic-related risk predictors	*N* (%)	*N* (%)	
Age (median, IQR)	80 (74–85)	81 (76–87)	0.97 [0.95;0.99]
Age ≥ 80 years	183 (51%)	258 (59%)	0.71 [0.54;0.94]
Male (vs. female)	177 (49%)	204 (47%)	1.10 [0.83;1.45]
Functional ability-related risk predictors	*N* (%)	*N* (%)	
Frailty (TRS tool)	217 (60%)	224 (51%)	1.28 [0.95;1.72]
Fallen previously (yes)	169 (48%)	203 (51%)	0.90 [0.67;1.20]
Immobility (yes)	167 (48%)	188 (47%)	1.02 [0.76;1.36]
Delirium, (4AT)-unlikely	125 (35%)	143 (36%)	–
Delirium, possible cognitive impairment	120 (34%)	150 (38%)	0.92 [0.65;1.29]
Delirium, possible delirium ± cognitive impairment	111 (31%)	105 (26%)	1.21 [0.84;1.73]
Disease-related risk predictors	*N* (%)	*N* (%)	
Chronic lung disease	33 (9%)	76 (17%)	0.48 [0.31;0.74]
*Primary presenting complaint at hospital admission*			
Respiratory disorders	63 (17%)	110 (25%)	0.63 [0.44;0.89]
Bleeding disorders	112 (31%)	12 (3%)	15.93 [8.61;29.48]
Gastrointestinal disorders	83 (23%)	36 (8%)	3.33 [2.19;5.06]
Syncope	37 (10%)	19 (4%)	2.51 [1.42;4.45]
*Charlson Comorbidity Index (CCI) (vs. none)*	57 (16%)	81 (19%)	–
CCI ≥ 1 and2 points	152 (42%)	183 (42%)	1.18 [0.79;1.76]
CCI ≥ 3	152 (42%)	172 (39%)	1.26 [0.84;1.88]
Medication-related risk predictors (ATC-code)	*N* (%)	*N* (%)	
Antithrombotic agents (B01)	294 (81%)	301 (69%)	1.98 [1.42;2.77]
Diuretics (C03)	184 (51%)	166 (38%)	1.70 [1.28;2.25]
Calcium channel blockers (C08)	109 (30%)	108 (25%)	1.32 [0.96;1.80]
Beta blocking agents (C07)	188 (52%)	207 (47%)	1.21 [0.91;1.60]
Psychoanaleptics (N06)	117 (32%)	142 (32%)	1.00 [0.74;1.34]
RAAS (C09)	205 (57%)	174 (40%)	1.99 [1.50;2.64]
NSAID (M01)	17 (5%)	34 (8%)	0.59 [0.32;1.07]
*Polypharmacy*			
Non-polypharmacy, ≤ 4 drug classes	46 (13%)	64 (15%)	–
Polypharmacy, 5–9 drug classes	164 (45%)	211 (48%)	1.08 [0.70;1.66]
Significant polypharmacy, ≥ 10 classes	151 (42%)	162 (37%)	1.30 [0.84;2.01]

53 individuals missing (at random) for Frailty (TRS)

*OR* odds ratio, *CI* confidence intervals, *IQR* inter-quartile ranges, *CCI* Charlson Comorbidity Index, *RAAS* renin–angiotensin–aldosterone system

### Development model and internal validation (ADAPTiP)

**Fig. 1:**
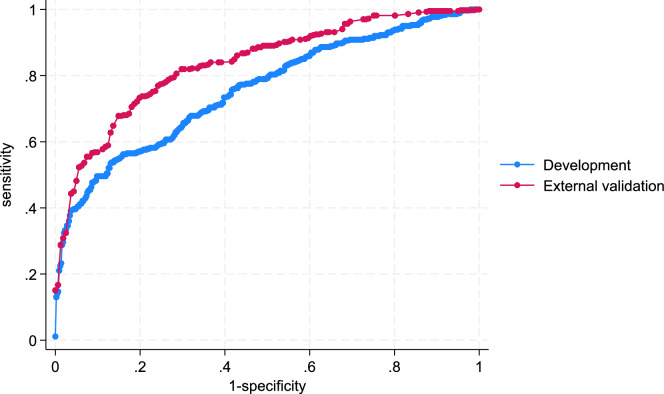
Discrimination in terms of cross-validated ROC curve for the ADAPTiP risk prediction model and ROC curve for external validation

Table 2 shows the final prediction model, ADAPTiP, which included nine predictors: age, chronic lung disease, the primary presenting complaints of respiratory disorders, bleeding disorders, gastrointestinal disorders and syncope on hospital admission and antithrombotic, diuretics, and RAAS drug classes. Presenting with a bleeding disorder contributed to the largest individual predictive value into the model [AUC 0.63; 95% CI 0.59;0.67], while presenting with syncope contributed to the lowest individual predictive value out of the nine included predictors [AUC 0.51; 95% CI 0.48;0.57]. Figure 1 displays the ROC curves for the final ADAPTiP model which showed clinically useful discrimination with a cross-validated AUC of 0.75 [95% CI 0.72;79]. The internal validation model showed adequate calibration (Supplemental Figure S1).

**Table 2:** Final ADAPTiP risk prediction model

	Univariate AUC and 95% CI	Stepwise AUC and 95% CI (without data split)	Stepwise AUC and 95% CI (cross-validated)	Multivariable coefficient and 95% CI
Bleeding disorders	0.63 [0.59;0.67]	0.64 [0.61;067]	0.63 [0.59;0.67]	17.43 [8.84;34.39]
RAAS	0.57 [0.53;0.61]	0.70 [0.67;0.74]	0.69 [0.65;0.72]	1.76 [1.27;2.44]
Gastrointestinal disorders	0.56 [0.52;0.60]	0.71 [0.67;0.74]	0.69 [0.65;0.72]	1.51 [0.86;2.66]
Antithrombotic agents	0.55 [0.51;0.59]	0.71 [0.68;0.75]	0.70 [0.66;0.73]	1.55 [1.05;2.28]
Age	0.55 [0.51;0.59]	0.73 [0.69;0.76]	0.72 [0.68;0.75]	0.96 [0.94;0.98]
Diuretics	0.54 [0.50;0.58]	0.74 [0.70;0.77]	0.73 [0.69;0.76]	1.75 [1.23;2.51]
Chronic lung disease	0.53 [0.49;0.57]	0.75 [0.71;0.78]	0.73 [0.70;0.77]	0.44 [0.26;0.73]
Respiratory disorders	0.52 [0.48;0.56]	0.75 [0.71;0.78]	0.73 [0.70;0.77]	1.30 [0.87;1.94]
Syncope	0.51 [0.48;0.57]	0.77 [0.73;0.80]	0.75 [0.72;0.79]	5.06 [2.70;9.48]

*AUC* area under the curve. Stepwise AUC reflects the predictive power of the model consisting of the corresponding variable as well as all above variables

### External validation of the model (ADAPTiP)

Table 3 presents the characteristics of the final ADAPTiP risk prediction model in the external validation dataset (CPCR). ADE is broader in scope (ADRs are a subset of ADEs) which accounts somewhat for the higher prevalence rate. In the external validation the AUC was 0.83 [95% CI 0.80;0.87]. The external validation model also showed adequate calibration (Supplemental Figure S2).

**Table 3:** Characteristics of the final ADAPTiP risk prediction model in the external validation dataset (CPCR) (*N* = 605)

Risk predictors	ADE *N* = 438	Non-ADE *N* = 160
Sociodemographic-related risk predictors	Median (IQR)	Median (IQR)
Age	79 (75–83)	78 (75–82)
Disease-related risk predictors	*N* (%)	*N* (%)
Chronic lung disease	55 (13%)	15 (9%)
*Presenting complaint to GP*		
Respiratory disorders	233 (53%)	38 (24%)
Bleeding disorders	262 (60%)	21 (13%)
Gastrointestinal disorders	146 (33%)	21 (13%)
Syncope	195 (45%)	24 (15%)
Medication-related risk predictors (ATC-code)	*N* (%)	*N* (%)
Antithrombotic agents (B01)	293 (67%)	62 (39%)
Diuretics (C03)	149 (34%)	27 (17%)
RAAS (C09)	207 (47%)	49 (31%)

IQR: inter-quartile ranges, RAAS: renin angiotensin aldosterone system. Data were missing at random for 7 patients

## Discussion

We have developed a risk prediction model, ADAPTiP based on nine predictors available from community-based older patients’ medical record at time of hospital admission, to help clinicians identify older patients who are at increased risk of an ADR/ADE. The model provided an AUC of 0.75 in internal cross-validation which is considered an acceptable discriminative capability for use in clinical practice [32]. ADAPTiP was externally validated in a cohort of community-dwelling older people attending general practice and achieved acceptable discriminative capability to identify ADE in the primary care setting.

ADAPTiP performs better than most of the previously developed risk prediction models, including the ADRROP model (AUC = 0.59) which was developed by combining four databases including 2217 older Irish people hospitalised with acute illness, the GerontoNET ADR Risk Score (AUC = 0.64) externally validated in a cohort of older adults hospitalised in Italy, the BADRI model (AUC = 0.74) developed in a population of older people from a UK teaching hospital, and the PRIME model (AUC = 0.69) which predicted medication-related harm (ADR was a subset of this definition) in the UK [33–36]. However, ADAPTiP does not perform as well as the PADROI tool (AUC = 0.89) developed in a referral hospital in south-western Uganda [37].

The predictors included in ADAPTiP were mainly determined based on their clinical relevance and ease of accessibility from patients’ medical records on hospital admission or in general practice. Other ADR/ADE risk prediction models do not necessarily contain measures that are easily accessible which may limit their use in clinical practice. The PADROI tool includes the use of potentially inappropriate medications (PIM), measured by the Beers 2019 criteria which consist of six different tables of PIM criteria to be considered within the context of the patients' medications and morbidities [38]. Both the PADROI and BADRI models include a length of hospital stay of ≥ 10 or ≥ 12 days, respectively, which is only applicable retrospectively to older patients who have been hospitalised (in-patients only). Other models such as the GerontoNET include whether or not the patient experienced a prior ADR, which may not be known given that primary and secondary care settings significantly under-report the incidence of ADRs/ADEs in older populations [39]. Many of the models also include laboratory results, such as renal failure, heart failure, liver failure, and white blood cell count (BADRI, PADROI, and GerontoNET [34, 35, 37]), and while these measures may be needed to accurately predict ADRs and ADEs, they may not be readily available in patients’ medical records and are difficult to generalise within the context of a generic ADR/ADE risk prediction tool.

The predictors included in the ADAPTiP model mainly consist of three drug classes, four primary presenting complaints, increasing age, and chronic lung disease. The drug classes (antithrombotic agents, diuretics, and RAAS agents) have all been shown to be implicated in ADRs and ADEs in older populations previously, and to cause potentially preventable admissions to hospital [40–42]. The primary presenting complaints of bleeding disorders, gastrointestinal disorders, and syncope are all indicators of innate toxicity in older populations, which may potentially be drug related [41]. Studies have indicated that the relationship between age and ADR/ADE is unclear with some studies showing older age to be associated with an increased risk, while others have found that the younger part of the elderly are more likely to experience ADR/ADE, because they are more likely to receive high-risk medications [33, 43]. In general, studies have not identified inhaled drug classes in those with chronic lung disease to be associated with ADR/ADE and requires further investigation [44].

ADAPTiP predicts well in a general practice setting using ADEs as the outcome of interest. It is essential that any developed ADR/ADE risk prediction model is generalisable to ‘similar but different’ individuals outside of the development cohort [45]. In general, the more the external validation setting differs from the development setting, the more robust the assessment of the generalisability of the model [15]. Expanding the validation across diverse geographic and healthcare settings would enhance the model's generalisability and appeal to a broader audience. There is a need for more generic ADR/ADE risk prediction tools that can be used across different countries and healthcare settings and allow for early detection of potential medication-related harm, e.g., mild ADEs that may develop into more significant adverse effects requiring hospitalisation. In fact, the previous research has indicated that 86% of patients in the CPCR cohort prescribed aspirin and warfarin reported bruising, bleeding, and abdominal pain [45], and these same drugs were found to be associated with 33% of ADR-related hospital admissions citing gastrointestinal and vascular haemorrhage disorders in the ADAPT cohort [17].

### Strengths and limitations

This study developed an ADR/ADE risk prediction tool, ADAPTiP, which has been internally and externally validated. A major strength of this prediction model compared to previous ADR/ADE prediction models is the investigational rigor and transparent reporting of the development and validation of the model as per the TRIPOD guidelines [11, 14]. It is also based on predictors which are easily assessable for clinicians. However, this study has some limitations. The development cohort is from a single large tertiary referral hospital and the external validation cohort consisted of older community-dwelling patients from one region of Ireland. The performance of ADAPTiP needs to be assessed across other healthcare settings, regions, and countries and with a larger number of patients experiencing ADRs/ADEs, to fully understand the transportability of the model and its clinical utility in practice. The identification and measurement of ADRs and ADEs are known to vary across studies and settings and such heterogeneity in measurement will affect model performance [46]. As described previously, while the determination of an ADR-related hospital admission in ADAPT included a multifaceted review of each potential ADR, including the use of a gold-standard medication reconciliation list and the application of a number of validated algorithms, there is the possibility of ADR misclassification given the difficulty of determining ADRs in older patients [17]. Similarly, despite a thorough review of ADEs by two independent clinicians, there may be misclassification. Future validation studies will need to consider this heterogeneity in the measurement of ADR/ADE outcomes to monitor and update the model as needed [47]. The model should also be assessed using the other forms of medication-related harm, such as rehospitalisation, functional impairment, and reduced quality of life.

### Future research

Further broader external validation of ADAPTiP across diverse healthcare settings is essential to demonstrate reproducibility and reliability, and it is the necessary first step towards implementation into clinical practice. To achieve robust external validation, TRIPOD guidelines recommend a head-to-head comparison between newly developed and existing ADR/ADE risk prediction models in an independent dataset [14]. The ADAPTiP model may need to be updated, adjusted or recalibrated, depending on performance in external validation studies. Following this, the impact of ADAPTiP on patient care and clinician behaviour needs to be analysed, ideally across both primary and secondary care settings [48]. This includes evaluating the acceptability of ADAPTiP to clinicians and patients, identifying potential barriers to implementation and how best it may be integrated into practice (potential incorporation into existing electronic healthcare systems) [49]. Comparison of ADAPTiP to clinical judgement may also aid clinician acceptability and use [50]. If the impact analysis demonstrates that ADAPTiP is effective and efficient and improves clinical care and patient outcomes, its cost-effectiveness should be evaluated to establish potential health care savings associated with routine use [48].

## Conclusion

Accurately predicting ADRs and ADEs in older populations is challenging given the numerous risk factors contributing to their occurrence, the difficulty in identifying and measuring ADRs/ADEs, and the heterogeneity of medical conditions in older populations [52]. ADAPTiP is a pragmatic risk prediction model, which, following further external validation, may help identify older patients at increased risk of medication-related harm, morbidity, and costs in our growing ageing population.

## Supplementary Information

Below is the link to the electronic supplementary material.

Supplementary Figure 1: Calibration plot of the ADAPTiP (cross-validated). Abbreviations: O:E: observed to expected ratio, CITL: calibration in the large, AUC: area under the curve. Interpretation of Supplementary Figure 1: O:E = 0.999. Very well calibrated since the observed to expected ratio is close to 1. The observed frequency of events closely matches the predicted frequency. This indicates that the model is not overestimating or underestimating the number of events. CITL = -0.003. The negative CITL value indicates that the average predicted probability is slightly higher than the actual observed frequency of the event. The model predicts slightly more events than actually occur in the data. However, the CITL is very close to 0 which means the model's overall predictions are very well-calibrated, with a very slight underestimation of the event frequency across the entire dataset. Slope = 0.920. A slope of 0.920 indicates that the model's predicted probabilities are slightly underestimating the likelihood of the event, but the calibration is still quite good. On average, the observed event frequency is only 92% of what the model predicted, which implies that the model is slightly overconfident as it predicts events too frequently compared to how often they actually occur. Supplementary Figure 2: Calibration plot of the ADAPTiP external validation model. Abbreviations: O:E: observed to expected ratio, CITL: calibration in the large, AUC: area under the curve. Interpretation of Supplementary Figure 1: O:E = 1.082. The model is underestimating the frequency of events. This is a relatively small miscalibration, indicating that the model is somewhat overconfident and predicts fewer events than actually happen in the population. CITL = 0.422. The CITL is a positive value, which means that, on average, the model is overestimating the likelihood of the event. CITL = 0.422 indicates a moderate degree of overestimation across all predictions. This is a relatively large miscalibration, which means the model is systematically predicting higher probabilities than what actually occurs. Slope = 0.817. The model is underestimating the likelihood of the event. Specifically, for every predicted probability, the model is predicting only 81.7% of the actual observed frequency of the event (DOCX 21 KB)

## Data Availability

The data that support the findings of this study are available from the corresponding author upon reasonable request. Some data may not be made available because of privacy or ethical restrictions.
